# Ferulic Acid, But Not All Hydroxycinnamic Acids, Is a Novel T3SS Inducer of *Ralstonia solanacearum* and Promotes Its Infection Process in Host Plants under Hydroponic Condition

**DOI:** 10.3389/fpls.2017.01595

**Published:** 2017-09-13

**Authors:** Yong Zhang, Jing Li, Weiqi Zhang, Rongsheng Wang, Qiaoqing Qiu, Feng Luo, Yasufumi Hikichi, Kouhei Ohnishi, Wei Ding

**Affiliations:** ^1^College of Resources and Environment, Southwest University Chongqing, China; ^2^The Ninth Peoples Hospital of Chongqing Chongqing, China; ^3^College of Plant Protection, Southwest University Chongqing, China; ^4^Laboratory of Plant Pathology and Biotechnology, Kochi University Kochi, Japan; ^5^Research Institute of Molecular Genetics, Kochi University Kochi, Japan

**Keywords:** *Ralstonia solanacearum*, type III secretion system, Hrp regulon, hydroxycinnamic acids, pathogenesis

## Abstract

Hydroxycinnamic acids (HCAs) are typical monocyclic phenylpropanoids, including cinnamic acid (Cin), coumaric acid (Cou), caffeic acid (Caf), ferulic acid (FA) and their isomers, and involved in the interactions between pathogens and host plants. Here, we focused on the impact of HCAs on expression of type III secretion system (T3SS) in *Ralstonia solanacearum*. FA significantly induced the expression of the T3SS and some type III effectors (T3Es) genes in *hrp*-inducing medium, while did not the other HCAs. However, exogenously supplemented FA did not affect the T3SS expression *in planta* and the elicitation of the hypersensitive response (HR) in tobacco leaves. Consistent with its central roles in pathogenicity, the FA-induced expression of the T3SS led to significant promotion on infection process of *R. solanacearum* in tomato plants under hydroponics cultivation. Moreover, the FA-induced expression of the T3SS was specifically mediated by the well-characterized signaling cascade PrhA-prhI/R-PrhJ-HrpG-HrpB, independent of the other known regulatory pathways. In summary, our results demonstrated that FA, a novel inducer of the T3SS in *R. solanacearum*, was able to promote its infection process in host plants under hydroponics condition.

## Introduction

As the causal agent of bacterial wilt, *Ralstonia solanacearum* causes serious loss in many economically important plants worldwide (Genin, 2010). *R. solanacearum* is a soil-dwelling, Gram-negative bacterium and can survive for days to years in plant materials and irrigation water (Janse et al., 2004). It generally enters host plants through root wounds or natural openings and rapidly colonizes the intercellular spaces of the root cortex, and eventually invades the xylem vessels (Roberts et al., 1988; Vasse et al., 1995). As vascular bacterium, the capacity of extensive colonization in xylem is essential for its virulence (Schell, 2000; Genin and Denny, 2012). In xylem vessels, the bacteria produce a large amount of extracellular polysaccharides (EPSs) slime, which ultimately blocks water flow and causes rapid stunting and wilting (Denny, 1995; Araud-Razou et al., 1998; Genin and Denny, 2012).

Like many Gram-negative bacteria, the syringe-like type III secretion system (T3SS) is essential for the pathogenicity of *R. solanacearum* in host plants. The bacteria use the T3SS to interact with host cells and inject virulence factors, so-called type III effectors (T3Es), into host cytosol to suppress the plant innate immunity and cause diseases (Cunnac et al., 2004; Angot et al., 2006; Jones and Dangl, 2006). The T3SS in *R. solanacearum* is encoded by approximately 20 hypersensitive response and pathogenicity (*hrp*) genes that are clustered together into a regulon (Galán and Collmer, 1999; Arnold et al., 2003). The *R. solanacearum* strains harbor a great number of T3E members and some of them are known to play important roles in the interactions with host cells (Coll and Valls, 2013; Peeters et al., 2013). *R. solanacearum* uses the master regulator HrpB, an AraC family transcriptional regulator, to directly control the transcription of T3SS and T3Es (Valls et al., 2006; Mukaihara et al., 2010). The expression of T3SS and T3Es is activated by plant signals or other related signals (Aldon et al., 2000). These signals are integrated by close paralogs, HrpG and PrhG. They are response regulators of the OmpR/PhoB family of two-component system, and positively regulate the *hrpB* expression in a parallel way (Plener et al., 2010; Zhang et al., 2013). *R. solanacearum* might use HrpG and PrhG to respond to host signals by phosphorylation at some residues (Cunnac et al., 2004; Yoshimochi et al., 2009b; Zhang et al., 2013). These signals are recognized by PrhA, an outer membrane protein, and transferred to HrpG through the well-characterized signaling cascade PrhA-PrhR/I-PrhJ-HrpG or some novel pathways (Brito et al., 2002; Cunnac et al., 2004; Valls et al., 2006; Yoshimochi et al., 2009b). Moreover, this signaling cascade is negatively regulated by the quorum-sensing dependent regulator PhcA via PrhIR, while PrhG is independent of this cascade and positively regulated by PhcA (Genin et al., 2005; Yoshimochi et al., 2009a; Zhang et al., 2013). *R. solanacearum* might switch from using HrpG to PrhG to *hrpB* activation in a cell density-dependent manner (Zhang et al., 2013).

The T3SS in *R. solanacearum* is not constitutively expressed under any condition. It is completely repressed in nutrient-rich media but well-induced in nutrient poor media, which might mimic conditions of intercellular spaces in host cells. Moreover, host signals or some related chemicals could induce the T3SS in *R. solanacearum* to a 10- to 20-fold higher level than that in nutrient-poor media (Marenda et al., 1998; Mukaihara et al., 2004; Yoshimochi et al., 2009b; Zhang et al., 2011). Some environmental factors (such as pH, temperature, nitrogen sources, and cell density) and plant related chemicals (typically plant phenolic compounds) could also affect (induce or inhibit) the expression of the T3SS in some phytobacteria (Tang et al., 2006; Stauber et al., 2012). It is reported that *t*-cinnamic acid and *o*-coumaric acid could induce the T3SS expression in phytobacterium *Dickeya dadantii* 3937 through the RsmB-RsmA pathway, while *p*-coumarate represses the T3SS expression through the HrpX/Y pathway (Yang et al., 2008; Li et al., 2009). Salicylic acid and benzoic acid could also inhibit the T3SS expression in phytobacterium *Erwinia amylovora* (Bailey et al., 2007; Felise et al., 2008). It is interesting that some exogenous compounds, which are the inhibitors of the T3SS, could impair the disease development of some phytobacteria in host plants (Khokhani et al., 2013; Yang et al., 2014). We recently performed a large-scale screening on root exudates of plant and identified some compounds as putative inhibitors or inducers of the T3SS expression in *R. solanacearum.* Among them we have characterized the oleanolic acid as a strong inducer of the T3SS. The oleanolic acid induced the expression of T3SS through the PrhA-HrpG-HrpB pathway (Wu et al., 2015).

**FIGURE 1:**
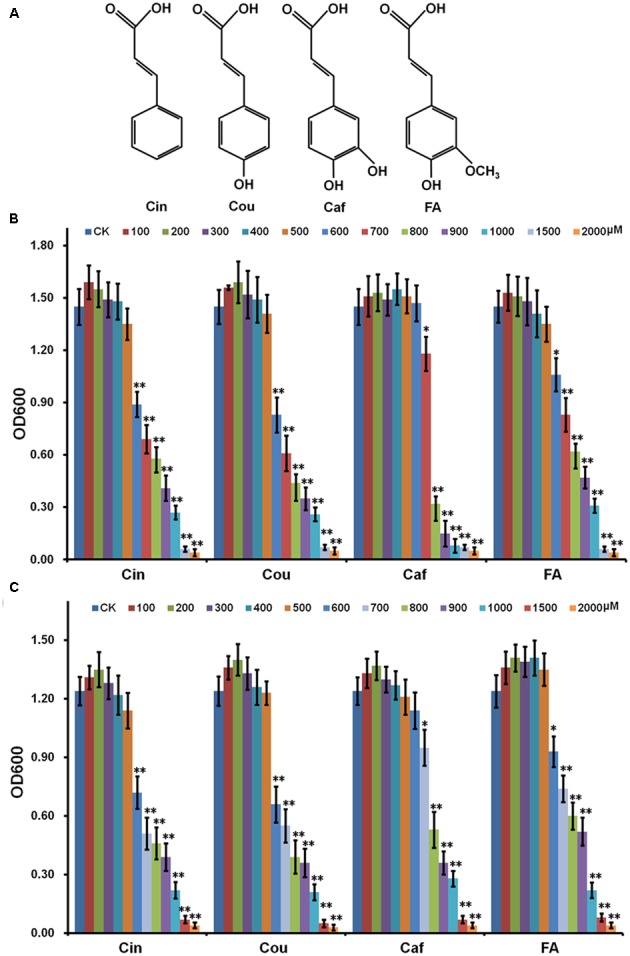
Effect of HCAs on growth of *Ralstonia solanacearum* in medium. **(A)** Structure of HCAs; **(B)** rich medium; **(C)** *hrp*-inducing medium. Cells were inoculated into fresh HCAs medium with the ratio as 1% and grown for about 16 h for OD_600_ measurement. HCAs were caffeic acid (Caf), cinnamic acid (Cin), *p*-coumaric acid (Cou), and ferulic acid (FA). Each test was repeated for at least three independent trials and each trial was repeated in twice. Mean values were averaged and presented with SD (error bars). Statistical significance between HCAs treatment and the control was assessed using a *post hoc* Dunnett test following ANOVA. Significance level, ^∗^ *p* < 0.05 and ^∗∗^*p* < 0.01.

Here, we focused on the Hydroxycinnamic acids (HCAs), which are monocyclic phenylpropanoid compounds with the C_6_–C_3_ skeleton. HCAs are hydroxyl derivatives of cinnamic acid, including cinnamic acid (Cin), coumaric acid (Cou), caffeic acid (Caf), ferulic acid (FA), and their isomers (**Figure 1**). HCAs are important precursors for lignin biosynthesis and several lines of evidence suggest that HCAs are involved in the interaction between plant pathogens and host plants (Fry et al., 2000; Naoumkina et al., 2010; Campos et al., 2014). Many plants could release *de novo* synthesized HCAs into the rhizosphere as response to root pathogens (Lanoue et al., 2010; Wallis and Chen, 2012). HCAs are broadly antimicrobial since they could disrupt membrane integrity and decouple the respiratory proton gradient (Fitzgerald et al., 2004; Harris et al., 2010). While *R. solanacearum* could protect itself from HCA toxicity by degrading low concentration of HCAs as sole carbon source. This degradation could also facilitate its disease development in host plants (Lowe et al., 2015). In this study, the impact of HCAs on the T3SS expression and disease development of *R. solanacearum* in host plants was investigated and its regulation was elucidated.

## Materials and Methods

### Bacterial Strains, Chemicals, and Culture Conditions

*Ralstonia solanacearum* strains used in this study were listed in **Table 1**. OE1-1 strain (phylotype I, race 1, biovar 3) is virulent in tomato and tobacco plants (Kanda et al., 2003). RS1002 strain (phylotype I, race 1, biovar 4) is virulent in tomato plants but elicit HR in tobacco leaves (Mukaihara et al., 2004). *R. solanacearum* was grown at 28°C in rich B medium or *hrp*-inducing medium (hydroponic plant culture medium containing 2% sucrose) (Yoshimochi et al., 2009b). Chemicals, primarily refer to Cinnamic acid (Cin), coumaric acid (Cou), caffeic acid (Caf), ferulic acid (FA) and DMSO were purchased from Sango Biotech (Shanghai, China). Stock solutions of HCAs were prepared in DMSO with appropriate concentrations and added into medium with the ration as 0.5–10% to get desired concentrations.

**Table 1:** *Ralstonia solanacearum* stains used in this study.

Strain	Relative characteristics	Reference source
OE1-1	Wild-type, race 1, biovar 3	Kanda et al., 2003
RK5043	OE1-1 *phcA-lacZYA*	Yoshimochi et al., 2009b
RK5046	OE1-1 *hrpB-lacZYA*	Yoshimochi et al., 2009b
RK5050	OE1-1 *popA-lacZYA*	Yoshimochi et al., 2009b
RK5120	OE1-1 *hrpG-lacZYA*	Yoshimochi et al., 2009b
RK5124	OE1-1 *prhJ-lacZYA*	Yoshimochi et al., 2009b
RK5130	OE1-1 *prhIR-lacZYA*	Yoshimochi et al., 2009b
RK5134	OE1-1 *prhA-lacZYA*	Yoshimochi et al., 2009b
RK5138	OE1-1 *xpsR-lacZYA*	Yoshimochi et al., 2009b
RK5212	OE1-1 *prhG-lacZYA*	Zhang et al., 2013
RK5283	OE1-1 *prhM-lacZYA*	Zhang et al., 2011
RK5613	OE1-1 *prhN-lacZYA*	Zhang et al., 2015b
RS1002	Wild-type, race 1, biovar 4	Mukaihara et al., 2004
RK10001	RS1002 *popA-lacZYA*	Zhang et al., 2011

### Measurement of Bacterial Growth of *R. solanacearum* in Medium

*Ralstonia solanacearum* was grown in rich B medium overnight and the bacterial cells were harvested. After washed with distilled water twice, bacterial cells were resuspended in distilled water with OD_600_ as 1.0 and inoculated into fresh medium with the ratio as 1%, and the bacterial grow from OD_600_ as 0.01. Bacterial growth was measured with OD_600_ after overnight (approximately 16 h) culture. Each assay was repeated for at least three independent experiments, and each treatment was repeated in twice. Mean values of all experiments were averaged and presented with standard deviation (SD). The statistical significance was evaluated using one-way ANOVA (GraphPad Prism 5, Version 5.01) in this study. After validating that datasets meet the ANOVA assumptions using the Bartlett’s test, D’Agostino and Pearson omnibus normality test, data from HCAs treatment were compared in parallel with those of control via a *post hoc* Dunnett test and the *p*-values were calculated.

### β-Galactosidase Assay

The gene expression level was evaluated by a *lacZYA* fusion-based β-galactosidase assay, in which the promoter-less *lacZYA* gene was fused to target genes and they shared the promoter (Zhang et al., 2013). The assay (both *in vitro* and *in planta*) was performed as previously described (Zhang et al., 2013). Each assay was repeated for at least three independent experiments, and each treatment was repeated in twice. Mean values of all experiments were averaged and presented with SD. The statistical significance between HCAs (or FA) treatment and the control was assessed using a *post hoc* Dunnett test following ANOVA.

### qRT-PCR Analysis

The OE1-1 was grown in *hrp*-inducing medium to OD_600_ as about 0.1 and total RNA was isolated using the TRIzol reagent method (Life, United States). After validating the RNA quality, cDNA was synthesized using the PrimeScript^TM^ RT Reagent Kit with gDNA Eraser (Perfect for Real Time, TAKARA, Japan) according to the manufacturer instructions (contaminated genome DNA could be completely removed by the gDNA Eraser in this kit). The One Step SYBR^®^ PrimeScript^TM^ PLUS RT-PCR Kit (TAKARA, Japan) was used for qRT-PCR reactions with Applied Biosystems 7500 Real-Time PCR System to quantify the cDNA level of target genes. Primers used in this study were selected as previously described (Wu et al., 2015) and the *serC* gene was used as reference gene for normalization of gene expression (Monteiro et al., 2012). Each assay was repeated from RNA isolation for at least three independent experiments, and each treatment included four replications. The mean values were averaged with SD, and the statistical significance between FA treatment and the control was assessed using a *post hoc* Dunnett test following ANOVA.

### Plant Cultivation, Virulence Assay, and HR Test

In this study, wilt-susceptible tomato plants (*Solanum lycopersicum* cv. Moneymaker) and tobacco plants (*Nicotiana tabacum* CV. Bright Yellow) were used for virulence test. Plants were cultivated at 25°C for 3–4 weeks and subjected for virulence assay with soil soak and petiole inoculation (Yao and Allen, 2007; Zhang et al., 2013). For the soil cultivation, plants were grown in plastic pot with humus soil (Pindstrup, Latvia) and irrigated with hogland medium every 3 days. For the hydroponic cultivation, plants were cultivated in sterile glass pot with hogland medium, and the medium was changed every 3 days. For the FA supplementation, hogland medium supplemented with 0.1 or 0.5 mM of FA was used for soil cultivation and hydroponic cultivation respectively. Each assay was repeated for at least four independent experiments and each treatment includes 12 plants. The mean values were averaged with SD, and the statistical significance between FA treatment and the control was assessed using a *post hoc* Dunnett test following ANOVA.

For HR test in tobacco leaves, *N. tabacum* BY leaves were infiltrated with bacterial suspension of RS1002 at 10^8^ cfu ml^-1^ by a blunt-end syringe (Zhang et al., 2015b). For the FA supplementation, bacterial suspension supplemented with 0.5 mM of FA was used for the leaf infiltration. HR symptom development was recorded periodically. Each experiment was repeated for at least three independent replicates, and a representative result was presented.

## Results

### Low-Concentration HCAs Were Non-toxic to *R. solanacearum* in Medium

Hydroxycinnamic acids are broadly antimicrobial and high-concentration HCAs are indeed toxic to *R. solanacearum*. Whereas the bacteria could protect itself from HCAs toxicity by degrading low-concentration HCAs as sole carbon source (Lowe et al., 2015). In this study, HCAs were added into medium in gradient concentrations and their effect on the growth of OE1-1 was investigated. High-concentration HCAs severely repressed the growth of OE1-1 in both rich medium (**Figure 1B**) and *hrp*-inducing medium (**Figure 1B**), while low-concentration HCAs didn’t affect its growth (**Figures 1B,C**). Without HCAs supplementation, *R. solanacearum* grew to an OD_600_ of 1.5 (± 0.12), that was identical to those with HCAs supplementation at 100–500 μM. In the case of Caf, the supplementation at 700 μM slightly repressed the bacterial growth to an OD_600_ of approximately 1.2 (*p* < 0.05, **Figure 1B**), whereas the supplementation at 800 μM significantly repressed the bacterial growth, corresponding to an OD_600_ of approximately 0.6 (*p* < 0.01, **Figure 1B**). Moreover, 600 μM of FA slightly repressed the bacterial growth (*p* < 0.05, **Figure 1B**), while 700 μM of FA significantly repressed the bacterial growth, corresponding to an OD_600_ of approximately 0.8 (*p* < 0.01, **Figure 1B**). For Cin and Cou, the supplementation at 600 μM significantly repressed the bacterial growth, corresponding to OD_600_ of approximately 0.8 (*p* < 0.01, **Figure 1B**). We also investigated heir effect on the growth of OE1-1 in *hrp*-inducing medium (**Figure 1C**), that was consistent with that in rich medium (**Figure 1B**). These results were consistent with previously reported results by Lowe, in which the strain GMI1000 was used (Lowe et al., 2015). The significant inhibitory concentration of HCAs were confirmed as follow: Caf, 800 μM; Cin, 600 μM; Cou 600 μM; FA, 700 μM. And hence, 500 μM of each HCAs were selected in this study for subsequent experiments.

### FA Significantly Induced the Expression of T3SS and Some T3Es in a Concentration-Dependent Manner

**FIGURE 2:**
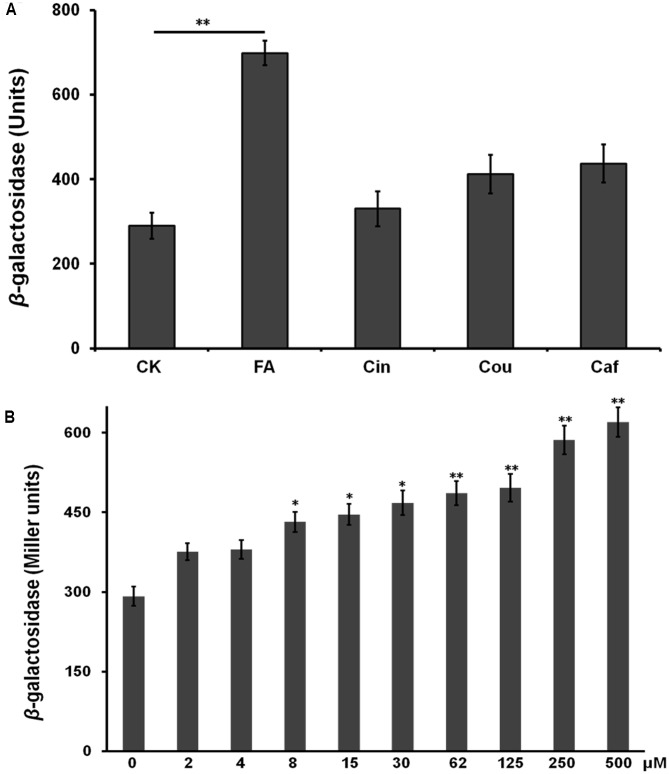
Effect of HCAs on the *popA* expression *in vitro.* **(A)** *hrp*-inducing medium supplemented with 0.5 mM of each HCAs; **(B)** *hrp*-inducing medium supplemented with gradient concentration of FA. Cells were grown in *hrp*-inducing medium to an OD_600_ of approximately 0.1 and subjected for β-galactosidase assay. The mean values of at least three independent trials are presented in Miller units with SD (error bars). Statistical significance between HCAs (or FA) treatment and the control was assessed using a *post hoc* Dunnett test following ANOVA. Significance level, ^∗^*p* < 0.05 and ^∗∗^*p* < 0.01.

Previously, we constructed a *popA-lacZYA* fusion to monitor the T3SS expression level in OE1-1 strain (Yoshimochi et al., 2009b), in which the promoterless *lacZYA* shared the promoter with *popA* (a schematic is available as Supplementary Figure S1 in Zhang et al., 2013). Each HCAs was added into *hrp*-inducing medium with the final concentration as 0.5 mM and their effect on the T3SS expression was investigated. Compared with the DMSO control (in *hrp*-inducing medium), FA induced the *popA* expression to approximately 2.0-fold higher level, while supplemented Cin, Caf, and Cou did not affect the *popA* expression (**Figure 2A**). In RS1002 strains, the supplemented FA could also significantly increase the *popA* expression, while supplemented Cin, Caf, and Cou did not affect the *popA* expression (data not shown), suggesting that FA-induced expression of the T3SS was not specific in the OE1-1 strain.

**FIGURE 3:**
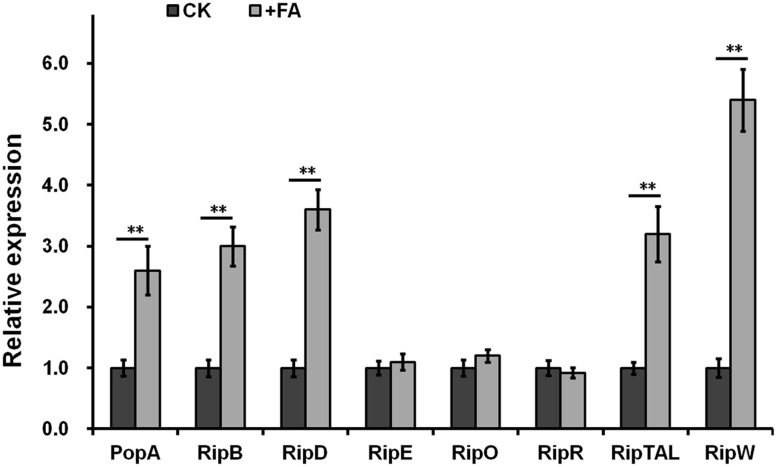
Relative expression of T3Es genes with FA treatment by qRT-PCR. The OE1-1 was grown in *hrp*-inducing medium (with 0.5 mM of FA or DMSO control) to OD_600_ as about 0.1 and total RNA was isolated for qRT-PCR analysis. Eight representative T3Es genes for selected for determination and the *serC* gene was used as reference gene for normalization of gene expression. Normalized value with FA treatment was divided with that of control and relative values were presented. Each test was repeated from RNA isolation in at least three independent trials and each trials included four replications. Mean values were averaged and presented with SD (error bars). Statistical significance between FA treatment and the DMSO control was assessed using a *post hoc* Dunnett test following ANOVA. Significance level, ^∗^*p* < 0.05 and ^∗∗^*p* < 0.01.

Some inducers usually exhibit higher inducing capacity on the T3SS expression at higher concentrations level, we further investigated the induction manner of FA at different concentration. FA was added into *hrp*-inducing medium with gradient increasing concentration (from 25 μM to 0.5 mM). The *popA* expression increased constantly with the increasing concentration of FA (**Figure 2B**), suggesting that the induction of FA on the T3SS expression was in a concentration-dependent manner. The *popA* gene exists as part of an operon with *popB* and *popC*, and belongs to T3Es. We further investigated whether FA could affect the expression of other T3Es. In this study, total RNA was isolated from OE1-1 strain and RipB, RipD, RipE, RipO, RipR, RipTAL, and RipW were selected for mRNA quantification by qRT-PCR, and the *popA* was used for positive control. Although FA treatment did not affect the mRNA levels of RipE, RipO and RipR, FA treatment significantly increased the mRNA levels of PopA, RipB, RipD, RipTAL, and RipW in *hrp*-inducing medium (more than twofold, *p* < 0.01, **Figure 3**).

### FA Induced the T3SS Expression through the PrhA-HrpG-HrpB Pathway

**FIGURE 4:**
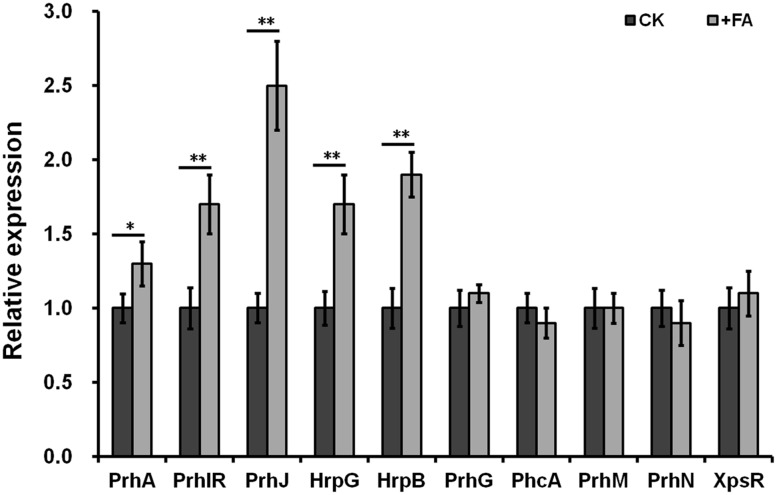
Relative expression of genes involved in *hrp* regulation with FA treatment. Cells were grown in *hrp*-inducing medium to an OD_600_ of approximately 0.1 and subjected for β-galactosidase assay. Normalized value with FA treatment was divided with that of control and relative values were presented. Each test was repeated in at least three independent trails and each trails included two replications. Mean values were averaged and presented with SD (error bars). Statistical significance between FA treatment and the DMSO control was assessed using a *post hoc* Dunnett test following ANOVA. Significance level, ^∗^*p* < 0.05 and ^∗∗^*p* < 0.01.

The T3SS expression in *R. solanacearum* was directly controlled by HrpB, and the *hrpB* expression is regulated by a complex regulation network. We therefore investigated whether FA induced the T3SS expression through some known pathway. We have generated several reporter strains from OE1-1 strain (promoter-less *lacZYA* was fused to these genes), including the *hrpB*, *hrpG*, *prhG*, *prhJ*, *prhIR*, *prhA*, *phcA*, *prhM*, *prhN*, and *xpsR* genes. The expression levels of these genes can be evaluated by the β-galactosidase assay. FA treatment could induce the expression levels of *hrpB*, *hrpG*, *prhJ*, *prhIR*, and *prhA* to approximately1.9-, 1.7-, 2.5-, 1.7-, and 1.3 -fold higher respectively. In contrast, FA treatment did not affect the expression of *prhG*, *phcA*, *prhN*, *prhM*, and *xpsR* (**Figure 4**). Therefore, FA induced the T3SS expression specifically through the PrhA-prhI/R-PrhJ-HrpG-HrpB pathway, and this induction is independent of other known regulators.

### Exogenously Supplemented FA Did Not Induce the T3SS Expression *In planta* and HR Elicitation

**FIGURE 5:**
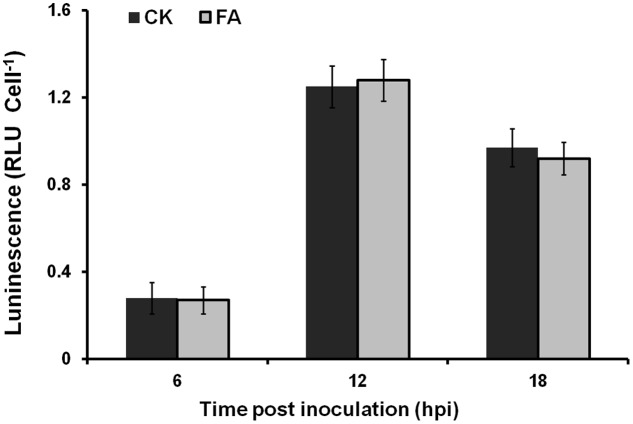
Effect of FA on the *popA* expression *in planta.* Gray bars, DMSO control; dark bars, FA treatment. Cells suspension of RK5050 (OE1-1 *popA-lacZYA*) with FA supplementation or DMSO control was infiltrated into tobacco leaves. Leaf disks were punched every 6 hpi and bacterial cells was collected for β-galactosidase assay. Each assay included at least three samples from different plants and mean values of at least four independent trails were averaged and presented in RLU cell^-1^ with SD (error bars).

The expression level of T3SS *in planta* could be induced to a much-higher level than that in *hrp*-inducing medium. We further investigated whether exogenously supplemented FA could induce the T3SS expression of OE1-1 to much-higher level *in planta*. Bacterial cells of RK5050 (OE1-1 *popA-lacZYA*) were resuspended in 0.5 mM of FA solution and infiltrated into tobacco leaves. Cells were collected from tobacco leaves and subjected for the β-galactosidase assay *in planta* using the Galacto-Light Plus kit. The *popA* expression of RK5050 in tobacco leaves was undetectable at time 0, and increased at 6, 12, and 18 hour post inoculation (hpi). While no difference in *popA* expression was observed in tobacco leaves between the FA treatment and the DMSO control (**Figure 5**), suggesting that supplemented FA could not induce the T3SS expression of OE1-1 to much-higher level *in planta*.

**FIGURE 6:**
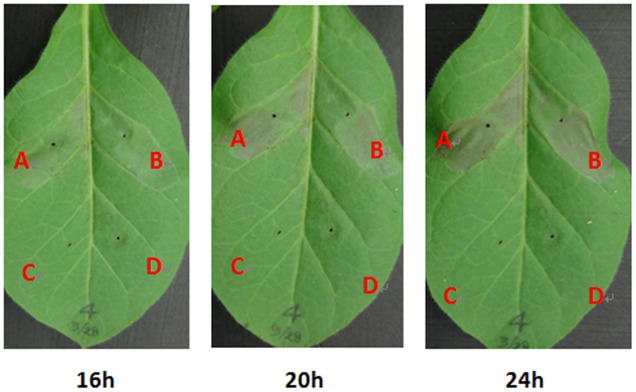
Effect of FA on HR elicitation of *R. solanacearum* in tobacco leaves. **(A)** Bacterial suspension with FA supplementation, **(B)** bacterial suspension with DMSO control, **(C)** FA solution, **(D)** DMSO control. Approximately, 50 μl of RS1002 bacterial suspension at 10^8^ cfu ml^-1^ was infiltrated into leaf mesophyll tissue with a blunt-end syringe. Pictures of tobacco leaves were taken at 16, 20, and 24 hpi. Each trail included at least seven plants and three independent trails were performed. Presented results were from approximately 20% (5 from total 21) tested tobacco leaves in which development of necrotic lesions was slightly faster than the that of control. Whereas no difference was observed in HR elicitation between FA treatment and the DMSO control in others 16 tested tobacco leaves.

Some T3Es are responsible for the HR elicitation in resistant plants, and we therefore evaluated whether supplemented FA could promote the HR elicitation in tobacco leaves. Bacterial cells of RS1002 were infiltrated into *N. tabacum* BY leaves and the development of necrotic lesions was investigated. FA treatment caused apparent necrotic lesions at 16 hpi, and the area of necrotic lesions reached a maximum at 24 hpi (**Figure 6**). It caused almost same HR elicitation as that of the DMSO control in most of the test tobacco leaves. Only in approximately 20% tested tobacco leaves (5 from total 21 leaves), the development of necrotic lesions with FA treatment was slightly faster than that of DMSO control (**Figure 6**), indicating that exogenously supplemented FA could not or just faintly promote the HR elicitation of RS1002 in tobacco leaves.

### FA Significantly Promoted the Infection Process of *R. solanacearum* in Host Plants, but Only under Hydroponics Condition

**FIGURE 7:**
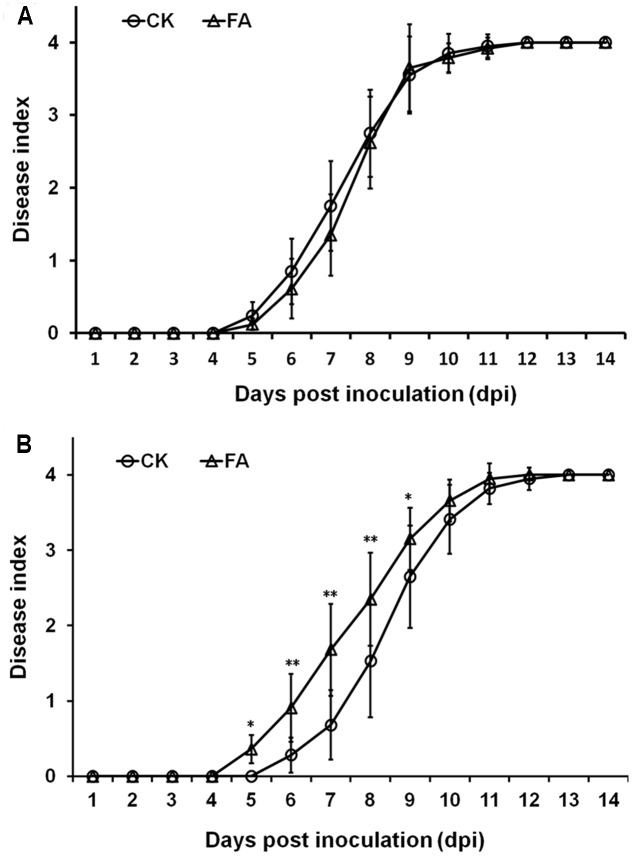
Effect of FA on infection process of *R. solanacearum*. **(A)** By soil-soak inoculation on humus soil cultivating tomato plants, **(B)** by root sipping inoculation on hydroponic cultivating tomato plants. Bacterial suspension was poured onto the humus soil or hydroponic medium at a final concentration of or 10^7^ cfu g^-1^ of soil or 10^7^ cfu ml^-1^ of medium. Opened circles, DMSO control; opened triangles, FA treatment. Plants were inspected daily for wilt symptoms, and scored on a disease index scale from 0 to 4 (0, no wilting; 1, 1–25% wilting; 2, 26–50% wilting; 3, 51–75% wilting; and 4, 76–100% wilted or dead). Each trail included at least 12 plants and mean values of at least three independent trails were averaged and presented with SD (error bars). Statistical significance between FA treatment and the control was assessed using a *post hoc* Dunnett test following ANOVA. Significance level, ^∗^*p* < 0.05 and ^∗∗^*p* < 0.01.

The T3SS and some T3Es play important roles in pathogenicity of *R. solanacearum* in host plants. Thus, we evaluated whether exogenously supplemented FA could affect the infection process of *R. solanacearum* in host plants. We supplemented FA into humus soil or hydroponic medium for plants cultivation and investigated the disease development. Under hydroponic cultivation condition, FA treatment caused obvious wilting symptom in root dipping inoculated tomato plants at 5 dpi. It is 1-day earlier than that of the control (**Figure 7B**, *p* < 0.05). At the stage of 5–8 dpi, the disease development with FA treatment was significantly faster than that of the control (**Figure 7B**, *p* < 0.01). FA treatment caused almost same disease development in tomato plants under hydroponic cultivation condition when plants were inoculated with petiole inoculation (**Figure 7A**). Under humus soil cultivation condition, FA treatment did not affect the disease development in tomato plants with either soil-soak or petiole inoculation (data not shown). It was suggested that exogenously supplemented FA could significantly promote the infection process of *R. solanacearum* in tomato plants under hydroponics condition but only when plants were inoculated with root dipping inoculation.

## Discussion

In the present study, we investigated the impact of HCAs on the T3SS expression in *R. solanacearum*. HCAs are broadly antimicrobial, and high-concentration HCAs can indeed severely repress the growth of *R. solanacearum* in medium. Whereas, *R. solanacearum* can protect itself from HCAs toxicity by degrading low-concentration HCAs as sole carbon source (Lowe et al., 2015). HCAs are hydroxy derivatives of cinnamic acid, with a C_6_–C_3_ skeleton. It is intriguing that only FA was found in this study to be able to significantly affect the T3SS expression in *R. solanacearum*. FA significantly induced the expression of the T3SS to much higher level *in vitro*. FA is 3-methoxy-4-hydroxycinnamic acid and the 3-methoxy in cinnamic acid is its main difference from other HCAs. Thus, this 3-methoxy in cinnamic acid should be responsible for its unique capacity on the induction of the T3SS in *R. solanacearum*. Some plant phenolic compounds and their derivatives have been characterized as inducer or inhibitor of the T3SS expression in some bacterial pathogens. For example, ferulate and some phenolics could induce the expression of some *Agrobacterium vir* genes; and flavonoids could induce the expression of *Rhizobium nod* genes (Bhattacharya et al., 2010). Moreover, some inducers exhibit higher capacity on the induction of the T3SS expression at higher concentration. It was consistent with our observation that the induction of FA on the T3SS expression in *R. solanacearum* was in a concentration-dependent manner.

Hydroxycinnamic acids are important precursors for the phenolic polymer lignin and are involved in the interaction with root pathogens. In response to root pathogens, many plants could release *de novo* synthesized HCAs into the rhizosphere, and accumulate HCAs or HCA-conjugates in their xylem sap (Mandal and Mitra, 2008; Lanoue et al., 2010; Wallis and Chen, 2012). For example, abundant FA and *p*-Cou are released from sentinel phenolic-storing-cells and accumulated in the xylem of tomato roots and stems when infected with the xylem-dwelling fungal vascular wilt pathogen *Fusarium oxysporum f.* sp. *Lycopersici* (Mandal and Mitra, 2008). Supplemented FA could induce the T3SS expression in *R. solanacearum* to much higher level than that in *hrp*-inducing medium alone, indicating that FA might mimic the host condition and thus activate the T3SS expression. Although HCAs concentrations required for growth inhibition *in vitro* are 10 to 100-fold higher than that measured *in planta*, HCAs concentrations in the xylem of roots and stems might be locally high where phenolics are released by sentinel phenolic-storing-cells (Beckman, 2000; Alvarez et al., 2008; Wallis and Chen, 2012). When host plants were exposed to *R. solanacearum*, abundant HCAs should be released and accumulated *in planta*. This could result in the locally high concentration of HCAs *in planta*. It is difficult for the exogenously supplemented FA to increase the T3SS expression *in planta* to much higher level than that with locally high concentration of HCAs. This explained our observation that no difference in *popA* expression was observed in tobacco leaves between the FA treatment and the control.

The T3SS plays essential roles in pathogenicity of *R. solanacearum* and supplemented FA could significantly induce the T3SS expression in *R. solanacearum*. Whereas, exogenously supplemented FA could promote the disease development of *R. solanacearum* in tomato plants under hydroponics condition only when plants were inoculated with root dipping inoculation. With the petiole inoculation, supplemented FA did not alter the disease development in tomato plants under either humus soil or hydroponic cultivating conditions. This might be due to the locally high concentration of HCAs in the xylem of roots and stems. The exogenously supplemented FA cannot increase the T3SS expression to much higher level *in planta*. Under condition of humus soil cultivation, supplemented FA did not alter the disease development in tomato plants with either soil-soak or petiole inoculation. Humus soil is the organic matter in the soil resulting from decomposition of plant and animal materials. Although we failed to examine the accurate concentration of FA in humus soil by HPLC, HCAs should be abundant in humus soil since HCAs are important precursors for the phenolic polymer lignin. At this condition, exogenously supplemented FA could not increase the T3SS expression any more. This could explain our observation that no promotion in disease development was observed in humus soil cultivating tomato plants. There was not any organic matter in hydroponic medium, and then supplemented FA could significantly improve the expression level of the T3SS in *R. solanacearum*. This resulted in the promotion on the disease development in tomato plants with FA treatment.

The expression of T3SS and T3Es is directly controlled by the master regulator HrpB and *hrpB* expression is regulated with a complex network (Arlat et al., 1992; Mukaihara et al., 2004, 2010). *R. solanacearum* strains have a large repertoire of T3Es (totally more than 90 T3Es), and there are averaged more than 70 T3Es per strain. Whereas, activation of HrpB does not result in the activation of all downstream T3Es genes in a given environment. Only about half of T3Es genes are up-regulated in wilting tomato plants compared to that in rich medium (Ishihara et al., 2012). And only a subset of T3Es from a given strain might contribute significantly to disease on a given host (Jacobs et al., 2012). This could explain our observation that supplemented FA could significantly induce the *hrpB* expression, but only five of eight test T3Es were significantly induced with FA treatment in *hrp*-inducing medium. We speculated that FA could induce the expression of more T3Es genes if more T3Es were tested. The activation of *hrpB* and T3SS expression needs plant signals or some mimic signals. These signals are recognized by the outer membrane protein PrhA and finally trigger the activation of *hrpB* expression through the PrhA-PrhR/PrhI-PrhJ-HrpG cascade (Valls et al., 2006; Hikichi et al., 2007). We previously demonstrated that some novel signal cascade was also involved in the *hrpB* expression, presumably by phosphorylation of HrpG with post-translational modification (Yoshimochi et al., 2009b). Moreover, the *hrpB* expression is also controlled by some important regulators such as PhcA, PrhG, PrhKLM, PrhN, and others (Zhang et al., 2011, 2015a). In this study, FA was revealed to induce the T3SS expression through the PrhA-HrpG-HrpB signal cascade, and this induction is independent of other known regulators such as PhcA, PrhG, PrhN, and others. Our finding is consistent with the biological function of PrhA. We previously reported that oleanolic acid could also induce the T3SS expression specifically through this PrhA mediated signal cascade.

Taken together, FA was characterized in this study as an novel inducer of the T3SS in *R. solanacearum* species. It significantly induced the T3SS expression in *hrp*-inducing medium through the PrhA-HrG-HrpB cascade, and could promote its infection process in tomato plants but only under hydroponics condition. This finding improves our understanding on T3SS regulators and various biological functions of plant phenolic compounds on regulation of the T3SS.

## Author Contributions

YZ, KO, and WD conceived and designed the experiments. YZ, JL, WZ, RW, and QQ performed the experiments. FL, YH, KO, and WD analyzed and discussed the results. YZ, JL, and KO wrote and revised the paper.

## Conflict of Interest Statement

The authors declare that the research was conducted in the absence of any commercial or financial relationships that could be construed as a potential conflict of interest.
